# Project SCORE (Student-Centered Outcomes Research Experience)

**DOI:** 10.3390/educsci16050745

**Published:** 2026-05-08

**Authors:** Marie Barnard, Tess Johnson, Allison Ford-Wade, Breanna Wade, Quest Whalen, Erin Dehon, Murrell Godfrey, Rachel Scott, Sarah K. Mason, Caroline E. Compretta

**Affiliations:** 1 Department of Pharmacy Administration, University of Mississippi, University, MS 38677, USA; 2 Department of Public Health, University of Mississippi, University, MS 38677, USA; 3 Department of Preventive Medicine, University of Mississippi Medical Center, Jackson, MS 39216, USA; 4 Department of Data Science, University of Mississippi Medical Center, Jackson, MS 39216, USA; 5 Department of Emergency Medicine, University of Mississippi Medical Center, Jackson, MS 39216, USA; 6 Graduate Shool and Department of Chemistry & Biochemistry, University of Mississippi, University, MS 38677, USA; 7 Center for Research Evaluation, University of Mississippi, University, MS 38677, USA

**Keywords:** public health education, STEM education, near peer mentoring, youth participatory action research, informal science education

## Abstract

Project SCORE (Student-Centered Outcomes Research Experiences) is a community-engaged after school science education program designed to address persistent inequities in health, education, and biomedical career access among Mississippi youth. Grounded in youth participatory action research and leveraging near-peer mentoring (NPM), the program engages teens in public health education, research skill development, and mentored inquiry led by undergraduate and graduate health sciences students. Program components include weekly workshops during the academic year and a one-week residential summer campus experience focused on health literacy, scientific thinking, research communication, and college-readiness. An evaluation assessed implementation and shortterm outcomes. Pre/post survey data indicate increases in STEM self-efficacy, career interest in STEM careers, and public health communication skills. Students reported strong engagement, belonging, and program satisfaction, and summer participants described an enhanced interest in college and health science careers. The lessons learned highlight the importance of robust NPM support, flexible program adaptation, and strong community partnerships. Early findings demonstrate that Project SCORE is a feasible, acceptable, and replicable model for engaging historically excluded youth in STEM and public health through community-based, student-centered research experiences.

## 1. Introduction

Systemic inequities in access to healthcare and quality educational opportunity for the youth of Mississippi (MS) are powerful forces that disengage students from entering pathways toward STEM careers. MS consistently ranks as one of the least healthy states in the United States. With some of the nation’s worst birth outcomes, highest rates of chronic diseases, and preventable mortality, Mississippians’ health status is poor (United Health Foundation, 2024). Poor health begins early. MS youth engage in significantly riskier health behaviors, including higher levels of tobacco use and risky sexual behaviors, and are significantly less likely to regularly report eating healthy food, exercising, and wearing a seat belt (Centers for Disease Control and Prevention, n.d.; Vargas & Zhang, 2017). Despite efforts by state and community agencies to promote healthy behaviors through educational and interventional programs, there has been little evidence that any of these initiatives have been effective.

The patient-centered approach, in which patients are “equitable partners–as opposed to research subjects–who leverage their lived experience and expertise to influence research to be more patient-centered, relevant, and useful” leads to an improved utilization of research results (PCORI Patient-Centered Outcomes Research Institute, 2018). A patient-engaged approach can be a valuable method to develop effective health behavior and promotion initiatives with adolescents. Given that existing health education, prevention, and intervention efforts for MS’s youth have been developed largely without input directly from youth, Project SCORE (Student-Centered Outcomes Research Experience) was developed to engage MS adolescents in a participatory action research effort designed to increase awareness of health disparities, promote health literacy and research engagement, and to expose students from historically excluded backgrounds to the wide variety of opportunities in the biomedical sciences.

Project SCORE centers the participants’ perspective by utilizing a youth participatory action (YPAR) approach, which enables youth to adopt a leading role in developing research projects focusing on issues that directly impact them (Anderson, 2019; Anyon et al., 2018; Lindquist-Grantz & Abraczinskas, 2020). YPAR operates under the premise that youth are experts on issues that directly impact them, and their unique viewpoint will improve the quality and relevance of the research. Prior research has shown that youth who have been directly engaged in YPAR develop research, leadership, and communication skills in addition to self-confidence (Anyon et al., 2018). Moreover, these youth are less likely to engage in unhealthy behaviors and are more likely to become advocates for healthy living (Anyon et al., 2018).

Near-peer mentoring (NPM) has gained attention as a way of engaging youth in research and increasing their interest in academic and research careers (Chester et al., 2018; Garcia-Melgar & Meyers, 2020; Jacquez et al., 2020). NPM by college and health profession-school students has been used successfully with underrepresented high school students in other STEM pipeline projects, including the NIH SEPA-funded West Virginia Health Science and Technology Academy (McKendall et al., 2014). Unlike traditional mentoring in which the mentor is an experienced faculty member, NPM utilizes students who are slightly more advanced in terms of education and training as mentors (Tenenbaum et al., 2014). NPMs can teach subject matter (e.g., scientific knowledge, research skills), provide career and academic guidance, and encourage mentees to pursue their goals. Further, recent reports indicate that NPM can provide important opportunities for interactions to address social and emotional issues for high school students (Qua et al., 2020).

The NPM approach has several advantages for both the mentee and the mentor. Mentees are better able to foresee themselves in the role of the mentors, given their closeness in age and educational stage to the mentor. Studies have found that mentees find it easier to learn from NPMs and perceive them as more “fun” and “easier to communicate with” compared to older mentors. NPMs also experience benefits from this approach. NPMs who are not yet subject matter experts gain the opportunity to advance their skills and content knowledge by teaching the mentee. NPMs reported gains in content knowledge, communication ability, and problem-solving skills after participating in NPM programs (Akinla et al., 2018; Trujillo et al., 2015). NPMs gain hands-on experience teaching and mentoring important skills for future scientists.

Near-peer mentors (NPM) have been shown to effectively support students in career exploration and research engagement (Garcia-Melgar & Meyers, 2020; Lee, 2019). Students who serve as mentors enhance their own knowledge and skills (Haggins et al., 2018; Trujillo et al., 2015). There is an urgent need for community-engaged research (CER) and immersing graduate students in this work prepares the next generation of CER scientists (Beaulieu et al., 2018; Boyer, 1996; DeLugan et al., 2014). Given that MS has one of the nation’s weakest STEM pipelines, with nearly the lowest rates of science and engineering bachelor’s degrees and individuals in STEM occupations, there is an urgent need to enhance interest in STEM (NSF—National Science Foundation, n.d.).

Project SCORE leverages the synergy of the YPAR and NPM approaches to engage students, expose them to public health concepts and careers, develop a student-centered health behavior and promotion research agenda, and participate in mentored research experiences. Through these aims, the project hopes to enhance health literacy, increase awareness and interest in careers in the health sciences with a focus on public health, encourage and provide direct guidance and support for students who wish to pursue higher education to prepare to participate in the biomedical workforce, provide near-peer mentoring to high school students from health sciences students who are specifically trained in effective mentorship procedures, train and mentor health sciences students in community-engaged research, increase their understanding of the scientific research process, and develop a student-centered health behavior and promotion research agenda. This paper describes the design and implementation of Project SCORE. We present our findings, examining the program feasibility, participant engagement and satisfaction, and preliminary changes in student-reported STEM self-efficacy, health literacy, and interest in health science careers.

## 2. Materials and Methods

### 2.1. Logic Model

**Figure 1.**
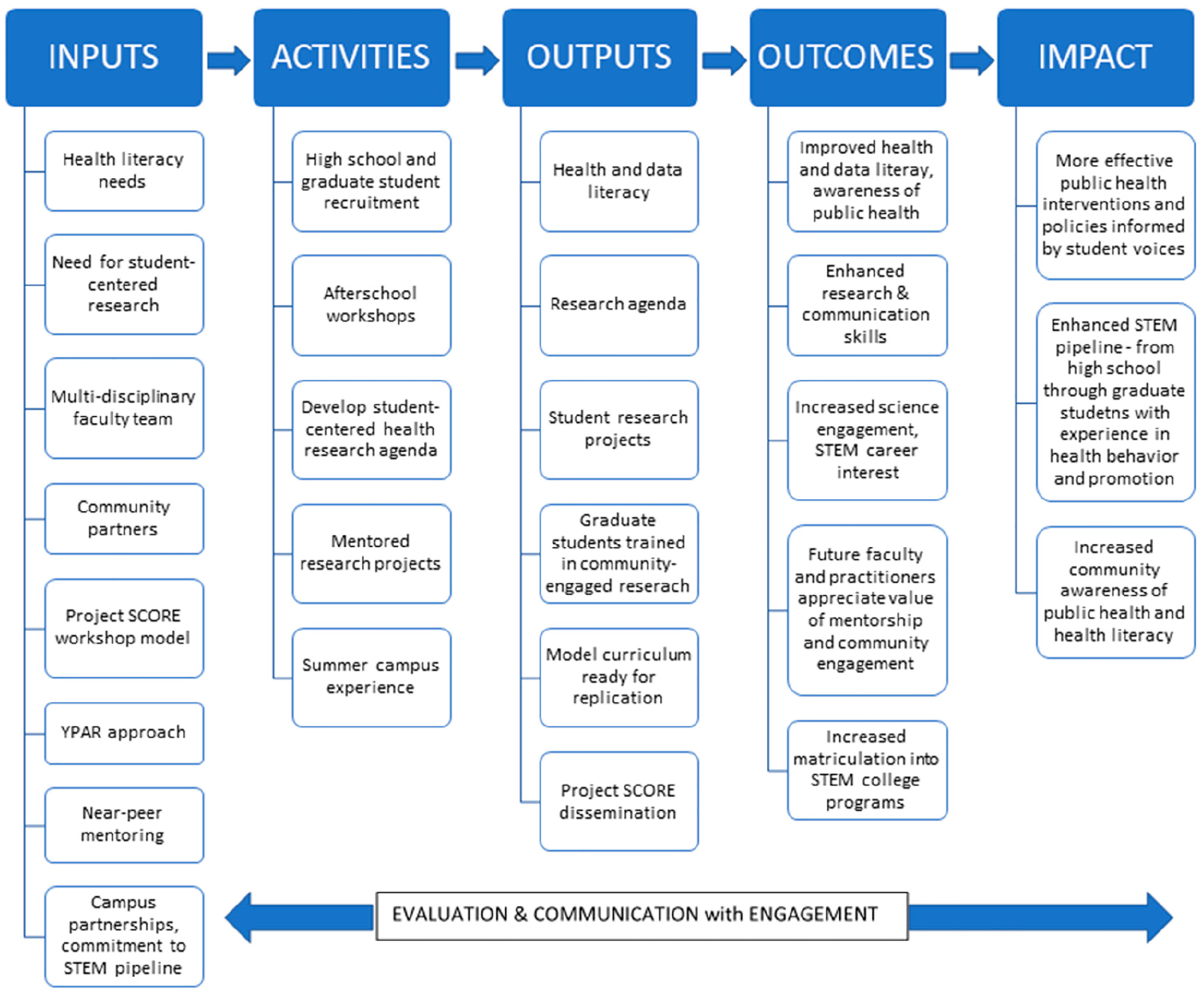
Project SCORE logic model.

The program operates with the following mission: As a comprehensive science enrichment program, we engage with students in participatory educational and research experiences in public health disciplines. Project SCORE aims to engage, educate, and mentor students to participate in the health sciences. This mission is accomplished with a comprehensive program that is captured in Figure 1, the logic model for Project SCORE. The model includes the program inputs and activities and describes the anticipated program outcomes and impact. The project is supported by a team of faculty and staff at the flagship public university in the state of Mississippi, local advisory boards at each site, and funding from the National Institute of General Medical Sciences. The project hypothesizes that participation in a year-long informal public health education program and mentored research experience utilizing a youth participatory action research approach and near-peer mentors will increase health literacy, science engagement, and interest in careers in the biomedical workforce; that graduate health sciences students who participate as near-peer mentors will value community-engaged research; and that a research agenda developed by youth will identify novel opportunities for health behavior and promotion research that will provide insight to develop efficacious community-relevant public health interventions.

### 2.2. Program Coordination

Project SCORE is managed by a faculty member and a full-time master’s level research associate in a school of pharmacy in a public university in the southeastern United States. Because the program operates at a second location in an urban environment nearly three hours away, there are also two faculty members and a part-time program manager based at the academic medical center campus who manage the second program site. Additional faculty collaborators are actively engaged in both sites.

### 2.3. Community Partnerships and Advisory Boards

Project SCORE is intentionally community-engaged. The project was developed in conjunction with two community partners who serve youth in the targeted communities. The Lafayette Oxford University (LOU) Boys and Girls Club is the Oxford community partner and Stewpot Community Service is the Jackson community partner. These two organizations have ongoing engagement with and programs for teens. Both of these community partners provide services such as transportation as part of their regular services outside of Project SCORE. They are integral to recruiting high school participants and host the academic year programming in their spaces.

### 2.4. Near-Peer Mentors

Undergraduate and graduate health sciences students are recruited by the program faculty to serve as SCORE Research Fellows. University students commit to lead the weekly workshops, to serve as near-peer mentors, and to collaborate with the high school SCORE Research Scholars on their research and health communication projects. The near-peer mentors participate in training that includes an introduction to community-based participatory and youth participatory action research, training them on how to serve as a mentor, responsible conduct of research, and other topics that are essential to their role. In addition, they have the opportunity to develop new networks of peers and of faculty mentors for themselves. The university students serving as NPMs are directly supervised by the program faculty and are mentored to support their professional development. The program faculty explicitly recognize that community-engaged research is challenging and provide support not only for the technical aspects (e.g., how to plan a session, how to support a research project), but also for the “power skills” necessary to function and succeed in a highly dynamic environment. NPMs participate in weekly training and de-briefing sessions to provide support and guidance in as close to real time as possible. Because the NPMs come from different disciplines, they bring different knowledge with them. This facilitates learning from each other, as well as from faculty, and builds a network of health science peers that they can carry forward in their careers.

### 2.5. Program Components

#### 2.5.1. Academic Year Program

Eight weekly after-school workshops are offered each semester for the high school Project SCORE Scholars. These workshops take place in the community partners’ facilities. In addition to a focus on preparing the students to engage in citizen science research, the program includes college and career development activities. These include an overview of the health sciences professions and research careers, mentoring by near-peer graduate health sciences students and faculty, and activities to develop critical thinking skills, health and data literacy, and presentation skills. Workshops follow a structured format each week: a welcome, a physical activity such as walking or yoga, an ice breaker activity to build community, a mindfulness activity, recall from prior sessions, and the introduction of a new lesson. The weekly workshop topics are included in Table 1. Importantly, workshops are led by the NPMs, with faculty present and available to assist as needed. This model facilitates the ability of the high school students to see the NPMs as leaders and role models.

#### 2.5.2. Summer Campus Program

Each summer, Project SCORE Scholars participate in a one-week residential campus experience. The goal of the SCORE Summer Campus Program is to expose students to a college environment and college faculty, and to engage in programming designed to further enhance their interests around education, research, and the STEM fields fostered by a diverse group of faculty and graduate students. Support to remove common barriers, such as transportation, luggage, and other items needed to live in a residence hall, are provided.

Students participate in a range of activities during the day. Programming includes a mix of engagement in academic activities, activities to make explicit the ‘hidden curriculum’ of college, and activities to facilitate interactions with university faculty and students. Students participate in presentations about university programs related to success in navigating how to gain admittance to and fund college, tours and meetings with offices such as campus housing, dining, and the bookstore to address hidden curriculum challenges, as well as the resources available to students for when they need assistance (e.g., the counseling and health centers, the Center for Students’ Success and First-Year Experience). Exposure to each office is followed by an engagement activity to reduce stigma associated with asking follow-up questions.

The camp is intentionally faculty-led to facilitate high school students’ interactions with health sciences faculty. Students are also exposed to campus resources and faculty associated with health sciences, such as the Health Professions Advising Office, to learn about academic pathways to various health professions and meet the admissions officers for health-related programs on campus. Students have small group meetings with faculty and graduate students in the health sciences to learn about their fields. Exposure to health sciences research activities includes lab tours and activities within some labs. For example, in the pharmacy skills lab, students participate in hands-on pharmacy skills. In the evenings, the participants engage with high school students participating in other university academic camp programming in structured, supervised activities. This exposes SCORE Scholars to a diverse group of peers and provides them with the opportunity to navigate a college environment with support.

### 2.6. Recruitment and Selection

High school students in grades 9–11 are recruited from the students who participate in the two community partners’ programs. Additionally, flyers advertising the program are distributed in local high schools. In-person information sessions are offered at both sites to provide prospective students and their families/caregivers with information about the program and to answer any questions they may have. Students who are interested in participating complete a brief application describing why they are interested in participating and indicate a commitment to attend the program regularly. When there are more applications than slots available, priority is given to students based on their academic year, with a preference for students in grade 11, followed by grades 10 and 9. Students who are not selected are encouraged to participate the next year and are not asked to reapply. The applications are reviewed and students are then officially offered a slot in the program as a SCORE Scholar.

### 2.7. Program Evaluation

The purpose of the evaluation is to provide feedback on program implementation so that it can be used for ongoing adaptation and improvement throughout the life of the program, to provide evidence for short- and long-term student outcomes for accountability purposes, and to contribute to the broader literature about the relationship between YPAR and students’ engagement in STEM (generally) and public health (specifically). The evaluation is guided by the logic model. Evaluation data collection includes a pre/post survey with the participating high school students. This project was approved by the University of Mississippi Institutional Review Board (22x-262). Students completed an informed consent form prior to data collection.

High school student participants complete pre- and post-surveys each year. Surveys were administered at the first and last session each year by the external evaluators. Students could opt to complete the survey on paper or online via Qualtrics. Student survey responses are incentivized using a $15 gift card. Student outcomes are assessment tools that have been previously validated with students of similar ages. The areas assessed include career interests (Tyler-Wood et al., 2010), self-efficacy in STEM (Baldwin et al., 1999), health literacy (Manganello et al., 2015), public health communication (Baldwin et al., 1999), and mindfulness (Greco et al., 2011; Prenoveau et al., 2018). On the post-survey, a sense of belonging in the program (Anderson-Butcher & Conroy, 2002; Skinner et al., 2009) and program engagement, utilizing an assessment created for this program, are also measured. Items’ means were calculated. Paired *t*-tests were calculated to compare pre and post measures. Table 2 provides more information on each outcome of interest that is assessed via the surveys.

## 3. Results

### 3.1. Participant Characteristics

Project SCORE aimed to recruit 10–15 teens each year at each of the two sites. When enrollment in the teen programming at one of the community partners decreased, Project SCORE added a community partner, the Boys & Girls Club of Central Mississippi, to increase participation. Participants are representative of the gender and racial/ethnic background of the students who participate in the community partner programming. Project SCORE met its recruitment goal, as teen participant enrollment included 109 total students from 2021 to 2025. The near-peer mentors have been drawn from a variety of academic programs across both institutions. This diverse group of university students has engaged with the teen participants in both the academic year and summer program. The participant demographics are described in Table 3.

### 3.2. Student Outcomes

Data are reported in Table 4 for students who agreed to participate in the surveys and completed both the pre and post survey from 2022 to 2025. Of the students who completed both surveys, attendance at all sessions was high. These participants missed no more than one session across the program year in which they participated. While there were no statistically significant differences between the pre and post assessments, participants reported improvements in STEM self-efficacy, career interests in STEM, and public health communication skills. A small decrease was reported in health literacy from pre to post assessment. Participants reported an improvement in mindfulness and a strong sense of engagement and belonging while participating in Project SCORE.

### 3.3. Programmatic Satisfaction

#### 3.3.1. Academic Year

**Figure 2.**
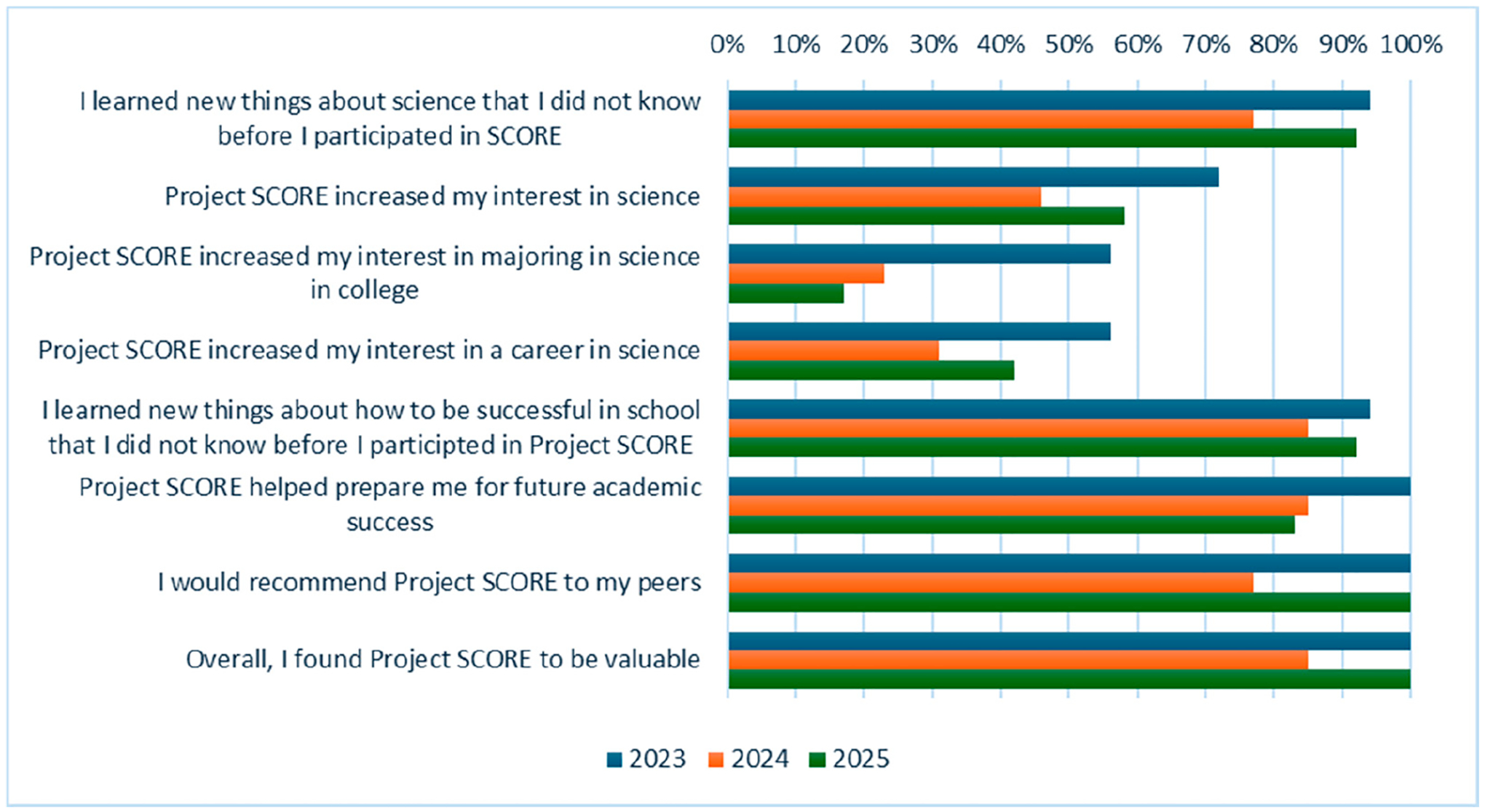
Post-program participation program evaluation (% agree/strongly agree).

At the end of each academic year, students were asked to respond to items related to their satisfaction with and perceived impact of the program. A subset of the students completed the post-program evaluation each year (2023 *n* = 18; 2024 *n* = 13; 2025 *n* = 12). The majority of the students agreed or strongly agreed that the program helped to prepare them for future academic success and that they learned new things about science. Students also reported that they would recommend the program to their peers and that they found participating valuable. Some of the students indicated that participation increased their interest in science and careers in science (Figure 2).

#### 3.3.2. Summer Camp

**Figure 3.**
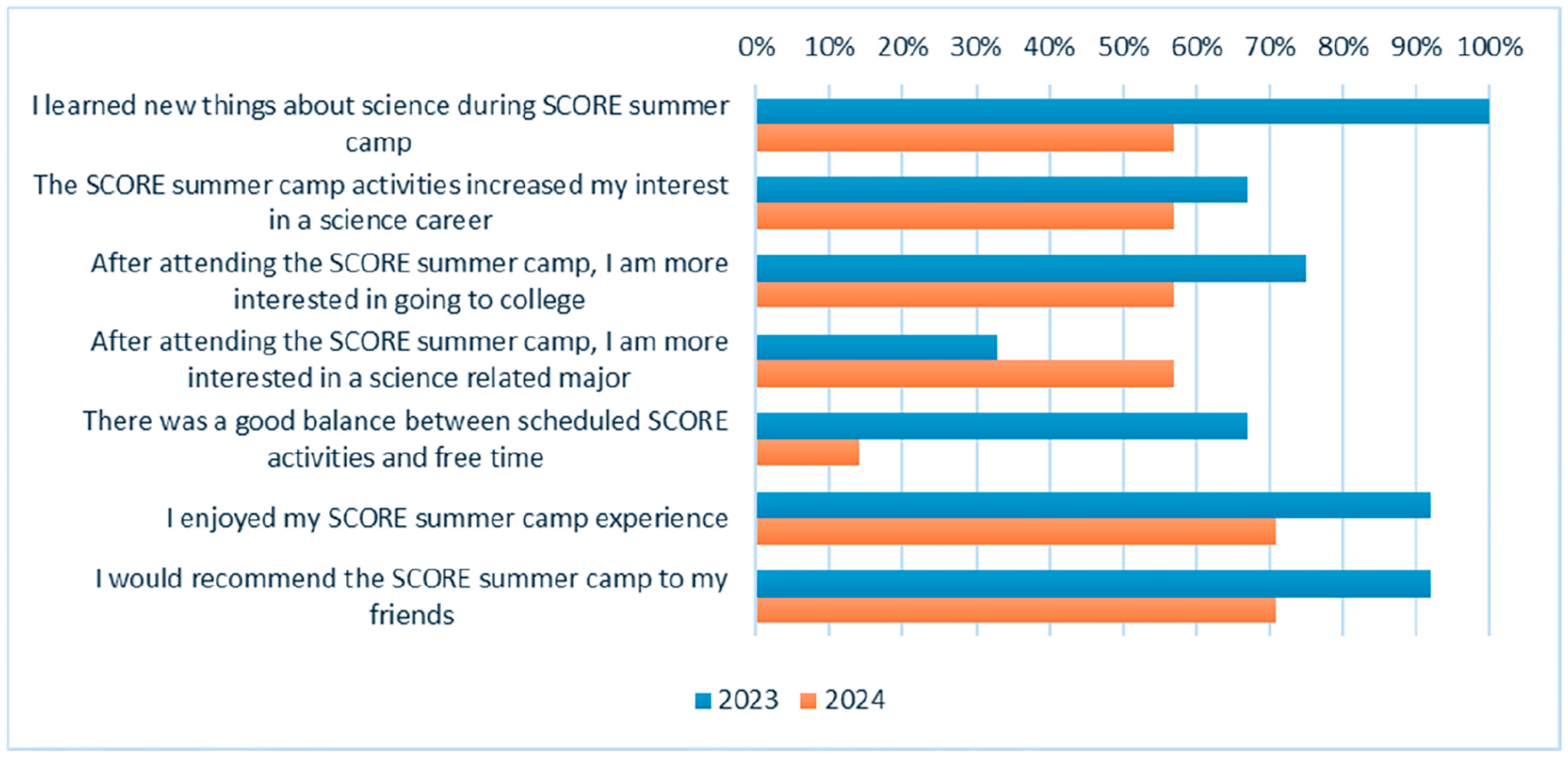
Project SCORE summer camp participant feedback (% agree/strongly agree).

A subset of the high school students come to the university campus each year for the week-long summer camp experience. All students who participated in the program during the academic year were invited to participate; however, students had individual family, school, and work commitments that did not allow for some of them to participate in the summer camp component. During the first year, no evaluation data were collected. Beginning with the second year of the camp program, students were asked to complete a brief post survey indicating their agreement with several statements. In both years, 100% of the summer camp participants completed the survey (2023 *n* = 12; 2024 *n* = 7). The responses indicated that the majority of students enjoyed the summer camp experience and learned new things about science. More than half of the students reported that participating in the camp increased their interest in going to college and in a career in science (Figure 3).

### 3.4. Areas for Continued Program Improvement

The data from the annual evaluation was utilized to improve programming each year. Adjustments made included adding training for the near-peer mentors, such as certification in mental health first aid, as well as adding more hands-on activities. Engagement in sessions in which the majority of activities were hands-on was consistently higher. The lack of improvement in health literacy scores has driven program changes to provide more practice in comprehending and applying health-related information. For example, we introduced an ice-breaker activity in which a statement a healthcare provider might make to a patient (e.g., “Take this medicine twice a day with a meal but not with any dairy.”) was whispered to a student and the student repeated the statement quietly to the next student. This was repeated until it got to the last student, who reported what the statement was by the time it reached them. The challenges of how health information is conveyed from provider to patient to caregiver was discussed, along with potential solutions to ensure that accurate information is available. Another activity called “Trust it or trash it?” in which students learn to critically evaluate online health information was implemented and applied to real-world health information examples that the students found online.

## 4. Discussion

This study examined the feasibility, acceptability, and preliminary outcomes of Project SCORE, a community-engaged public health pipeline program that integrates youth participatory action research and near-peer mentoring. The findings suggest that the model is feasible to implement across community settings and highly acceptable for participants, as evidenced by strong engagement, sense of belonging, and program satisfaction. Although pre–post changes in student outcomes were modest, trends toward improved STEM self-efficacy and career interest align with the prior literature suggesting that sustained exposure to mentoring, applied STEM learning, and research participation may positively shape educational aspirations among historically underrepresented youth.

The strong levels of engagement and belonging observed among participants are consistent with the prior YPAR literature, which suggests that youth are more engaged when they are positioned as contributors to knowledge generation, rather than passive recipients of instruction (Anyon et al., 2018; Anderson, 2019). Project SCORE extends the literature by embedding YPAR within an informal science education and public health pipeline framework. While many YPAR programs focus primarily on civic engagement or issue advocacy, Project SCORE demonstrates how participatory research methods can also be leveraged to support STEM exposure and workforce development.

Findings related to participant engagement may also reflect the role of near-peer mentoring. Prior studies have found that near-peer mentors are often perceived as being more approachable and relatable than traditional adult mentors and can positively influence students’ academic confidence and STEM identity (Tenenbaum et al., 2014; Garcia-Melgar & Meyers, 2020; Qua et al., 2020). Our observations similarly suggested that undergraduate mentors were particularly effective at relationship-building with participants. This finding expands the prior literature by demonstrating the potential value of near-peer mentoring within community-based public health education programs.

Similar to other pipeline initiatives such as the West Virginia Health Sciences and Technology Academy (McKendall et al., 2014), Project SCORE seeks to address structural barriers to STEM participation among rural and historically excluded youth. However, Project SCORE differs from many traditional pipeline programs by explicitly integrating health literacy, public health communication, and youth-driven research agenda development. These features may be particularly important in communities experiencing persistent health inequities.

### 4.1. Lessons Learned

The Project SCORE team has learned throughout program implementation by utilizing ongoing process evaluation, team meetings, and session debriefs to identify barriers and facilitators and have moved to quickly adapt the program. One of the first lessons learned was that it helped to have more NPMs and project staff at each session than originally planned. Two NPMs for each site were originally planned, but we added three to five additional NPMs for each site, bringing the program to a two to three teens to one adult ratio. Adding team members allowed the program to provide more support for the high school students in the program. In the process of adding team members, we added some undergraduate students to join the project as NPMs. Undergraduate students were able to connect with the teens more easily, and based on this observation, we began to recruit undergraduate students as NPMs in future years. This adaptation was effective and we urge other programs to consider staffing at a level that supports developing relationships and providing more individualized support when working with teens. The university required substantial training in the protection of minors for all adults, including NPMs, working on the project and the team completed additional training in youth mental health first aid and CPR/first aid.

We also learned that it is challenging to recruit high school students to participate in after school programming. Although the program design includes providing a stipend for each session the students participate in, students dropped out due to the demand of after school jobs. Childcare responsibilities for siblings, sports activities, and church activities also impacted the participation levels. Programs should consider identifying local needs and provide the resources and support needed to facilitate participation in similar programs. Finally, Project SCORE operates in Mississippi and the state was hit hard by the COVID-19 pandemic, a water access crisis, and the impact of climate change. All of these issues delayed, canceled, and required adaptation of the program sessions. For example, in the second year, we adapted the first session team project, which was to build a CorsiRosenthal air cleaner as a way to introduce public health, to building a home-made water purifier at the Jackson site in response to the water crisis. Other adjustments included meeting twice a week when a previous week was canceled due to an increase in COVID-19 cases that closed the schools in the community, lengthening sessions when weather events required the students to stay at the community facilities for longer, and meeting students’ informational needs with regard to the public health crises that the communities were experiencing.

### 4.2. Next Steps

The findings reported here are based on the first four years of program implementation. Tracking of program participants, both of the high school SCORE Scholars and of the university-level SCORE Fellows, is planned to continue to assess the program’s impact. The student-developed research agenda is being developed for scientific dissemination by a team of the participating high school students, near-peer mentors, and program faculty. Program dissemination efforts are underway. For example, the program is currently being adapted to support the expansion of the program to incorporate middle school students. Given the financial resources needed to operate the summer camp program, it is unlikely that this can be continued without external funding support. However, the after school program could be implemented for a minimal cost if students are not compensated for participation.

### 4.3. Limitations

Project SCORE is relatively new, with only four years of program experience. Additionally, the program has only been operated in the state of Mississippi. High school program participants have not reached high school graduation, so program outcomes such as college matriculation, enrollment in STEM college majors, and career outcomes are not yet available. While 109 students have participated in the program, just under half (47%) completed both the pre and post survey. Some students chose not to complete the surveys, and others were not present for the first or last session. While the participation rate for the group that completed the pre and post surveys was high, participation varied substantially for those that did not complete both surveys. The students who are not included in the analyses were more likely to be have individual circumstances that prevented consistent participation. It is likely that these participants who received a small dose of the program would have reported less program engagement and would not have experienced any gains in self-efficacy, career interest, or public health communication skills. The results reported here represent findings for participants who received a full dose of the program. Additional research would be needed to understand the potential impact of more limited program exposure. Further, no comparison group was utilized for this investigation.

## 5. Conclusions

Findings from the first years of the program provide proof of concept that Project SCORE is one programmatic strategy to engage youth in learning about the field of public health, increase their ability to be successful in school, and increase interest in science. The Project SCORE curriculum manual with lesson plans, training plans, and other materials is available at https://egrove.olemiss.edu/ and we welcome others to utilize these resources to develop their own programs to engage youth in similar programming.

## Figures and Tables

**Table 1.** Academic year workshop topics.

Semester 1 Topics	Semester 2 Topics
Community building—orientation to the program	Developing a student-centered research agenda
Introduction to public health	Research methods and ethics
What is epidemiology?	Health education and health communication
Careers in the health sciences	Selecting a project
Introduction to health literacy	Project work (3 weeks)
Introduction to health disparities	Project presentations
Introduction to research and the scientific method	
Developing research questions	

**Table 2.** Descriptions of Outcome Measures.

Outcome	Description	Scaled Response
Self-efficacy in STEM	Students’ beliefs regarding their ability to engage in STEM learning	1 = Not Confident At All to 5 = Totally Confident *Higher scores indicate higher levels of self-efficacy*
Career Interest in Public Health	Students’ interest in jobs related to public and allied health, medicine, science, and research	1 = Strongly Disagree to 5 = Strongly Agree *Higher scores indicate higher levels of career interest*
Mindfulness	Students’ ability to observe internal experiences, act with awareness, and accept internal experiences without judging them	−1 = Never True to 5 = Always True *Lower scores indicate higher levels of mindfulness*
Public Health Communication	Students’ use of verbal and written strategies to effectively communicate health-related topics.	1 = Never True to 5 = Always True *Lower scores indicate higher levels of use of strategies*
Health Literacy	Students’ ability to find, understand, and make health-related decisions	1 = Rarely to 4 = Always *Higher scores indicate higher levels of health literacy*
SCORE Engagement^1^	Students’ interaction with and interest in SCORE workshops and activities	1 = Strongly Disagree to 5 = Strongly Agree *Higher scores indicate higher levels of engagement*
SCORE Sense of Belonging ^1^	Students’ feelings of acceptance, value, and inclusion in the SCORE program	1 = NO! to 4 = YES! *Higher scores indicate higher levels of sense of belonging*

1 SCORE engagement and SCORE sense of belonging are collected during post-survey only.

**Table 3.** Participant demographics.

	Teen Participants (SCORE Scholars) *N* = 109	Near-Peer Mentors (SCORE Fellows) *N* = 20
% Female	64.2%	70%
% African American Black	93.6%	60%
% White	3.7%	20%
% Other	2.8%	20%

**Table 4.** Pre and post comparison of program outcomes.

Outcome (*n* = Participants with Pre/Post Data)	Pre-Program Participation Item Mean (SD)	Post-Program Participation Item Mean (SD)
STEM Self-efficacy (*n* = 51)	2.89 (1.16)	3.06 (1.20)
Career Interest in STEM (*n* = 51)	3.41 (0.91)	3.47 (0.78)
Health Literacy (*n* = 49)	2.49 (0.73)	2.39 (0.60)
Public Health Communication^1^ (*n* = 49)	3.23 (1.07)	3.17 (0.84)
Mindfulness ^1,2^ (*n* = 24)	2.94 (0.76)	2.71 (0.68)
Sense of Belonging (*n* = 51)	N/A	4.10 (0.61)
Engagement (*n* = 51)	N/A	4.33 (0.71)

1 Lower scores indicate higher use of strategies.

2 Mindfulness was not collected in the first two years of the program.

## Data Availability

The archived dataset is available in https://egrove.olemiss.edu/ as Project SCORE data.

## Abbreviations

The following abbreviations are used in this manuscript:

NPM: Near-Peer Mentor
STEM: Science Technology Engineering and Math
MS: Mississippi
YPAR: Youth Participatory Action Research
SCORE: Student-Centered Outcomes Research Experience
CER: Community-Engaged Research
